# Cardiac timing effects on response speed are modulated by blood pressure but not heart rate variability in healthy young adults

**DOI:** 10.14814/phy2.70590

**Published:** 2025-09-29

**Authors:** Xiao Yang, Catalina Roldan, Michael Gazzanigo, Yasmine Nabulsi, Fang Fang

**Affiliations:** ^1^ Department of Psychology Old Dominion University Norfolk Virginia USA; ^2^ Research and Infrastructure Service Enterprise Virginia Health Sciences at ODU Norfolk Virginia USA

**Keywords:** blood pressure, cardiac timing effects, cognitive interference, interoceptive processes, vagally‐mediated heart rate variability

## Abstract

Cardiac timing effects reflect the dynamic interplay between interoceptive and exteroceptive processes. Human information processing tends to be facilitated during cardiac diastole and inhibited during systole, reflecting autonomic regulation and the neuromodulation by baroreceptor afferents. Thus, blood pressure (BP) and vagally‐mediated heart rate variability (vmHRV) are potential modulators of those effects. Additionally, cognitive control appears to play a critical role in the processes. However, whether cardiac timing effects are influenced by those factors remains unclear. The present study aimed to clarify these relationships. Fifty‐one healthy young adults completed three experimental sessions to assess BP, short‐term HRV, and cardiac timing effects. The Multi‐Source Interference Task served as the cognitive task, with stimuli presented during either systole (R + 300 ms) or diastole (R + 550 ms). Repeated‐measures ANCOVA and regression analyses were conducted to examine the effects of cardiac timing on response time (RT) and their associations with BP and vmHRV. Results indicated that higher BP predicted less RT slowing by systole in interference trials, while vmHRV was not linked to cardiac timing effects in interference or control trials. These findings suggest that individual differences in physiological functioning influence cardiac timing effects and contribute to better understandings of how interoceptive processes shape human cognition.

## 1 INTRODUCTION

Human information processing is embodied, involving dynamic interactions between interoceptive and exteroceptive processes (Yang et al., 2017). Cardiovascular afferent input serves as a key interoceptive mechanism through which bodily states influence cognitive processes (for reviews, see Critchley & Garfinkel, 2015; Schulz & Vögele, 2024). According to Lacey and Lacey's hypothesis (1978), afferent signals generated by the distension of arteries due to pulsatile blood flow—primarily during cardiac systole—induce cortical inhibition. This effect is mediated by baroreceptors, mechanoreceptive sensory neurons located in blood vessel walls that detect changes in blood pressure. During systole, blood is ejected into the aorta, activating baroreceptors, whose signals are transmitted to the nucleus tractus solitarius (NTS; Henderson et al., 2004). Subsequently, the neural output from the NTS inhibits cortical processing of external stimuli. In contrast, during diastole, baroreceptor firing decreases, facilitating environmental information processing (Lacey & Lacey, 1970, 1978). The ongoing fluctuation of interoceptive states allows for continuous adaptation to environmental demands and supports the efficient allocation of resources for perception, cognition, and behavior (Duschek et al., 2013).

Cortical inhibition elicited by cardiac afferents is reflected in cardiac timing effects on cognitive performance. Specifically, information processing tends to be more efficient when stimuli are presented during cardiac diastole compared to systole (Critchley & Garfinkel, 2015). A common method to study these effects involves time‐locked stimulus presentation to specific phases of the cardiac cycle using electrocardiography (ECG) signals (Edwards et al., 2007, 2009). Baroreceptor activation typically occurs between 90 and 390 ms after the R‐wave, with peak output lasting around 250 ms (Edwards et al., 2009). Consequently, cognitive task stimuli are often delivered at approximately 300 ms (systole) and 550 ms (diastole) post‐R‐wave (e.g., Izaki et al., 2024; Rae et al., 2018; Schulz et al., 2009; Yang, Chaney, et al., 2024; Yang, Herberlein, et al., 2024). Although this method faces limitations in precisely identifying cardiovascular events (Caparco et al., 2025), accumulating evidence shows performance differences across cardiac phases. Particularly, response speed, as measured by reaction time (RT), is typically faster during diastole (Birren et al., 1963; Callaway & Layne, 1964; McIntyre et al., 2008), and RT appears to vary linearly across portions of the cardiac cycle (Stewart et al., 2006).

Cardiac timing effects have been observed across a wide spectrum of cognitive domains in laboratory studies, including attention (Kunzendorf et al., 2019; Li et al., 2018; Ren et al., 2022), sensorimotor and affective processing (Garfinkel et al., 2014; Saltafossi et al., 2023; Yang et al., 2017), memory (Fiacconi et al., 2016; Garfinkel et al., 2013), cognitive control (Larra et al., 2020; von Haugwitz et al., 2024; Yang et al., 2023; Yang, Herberlein, et al., 2024), decision‐making (Herman & Tsakiris, 2020; Kimura et al., 2023), learning (Pfeifer et al., 2017), and social cognition (Azevedo et al., 2017; Izaki et al., 2024). However, some studies have reported null or conflicting results (Salzman & Jaques, 1976; Thompson & Botwinick, 1970; Weisz & Ádám, 1996), and the nature of cardiac timing effects appears to vary depending on the cognitive task (Adelhöfer et al., 2020; Larra et al., 2020; Yang, Herberlein, et al., 2024). As noted by Carroll and Anastasiades (1978), a variety of contextual and methodological factors may influence how cardiac timing effects manifest in specific experimental settings.

Given that cardiac timing effects reflect baroreceptor‐mediated neuromodulation (Lacey & Lacey, 1978), blood pressure (BP) status is a critical factor influencing these effects. Schulz et al. (2011) found that elevated BP during a laboratory‐induced stressor altered the pattern of cardiac cycle modulation of the startle response, compared to a nonstress control condition. Specifically, under high BP—particularly elevated systolic BP—the trough and peak of startle reactivity at systole and diastole occurred earlier following the ECG R‐wave. In that study, stimuli were delivered at multiple time points across the cardiac cycle; notably, when comparing responses at R + 300 ms (systole) and R + 500 ms (diastole), the magnitude of the cardiac timing effect was reduced under high BP conditions. However, trait differences in BP status were not examined in relation to cardiac timing effects in the study (Schulz et al., 2011). Also, the startle response is enhanced by cardiac systole, which is different from the cardiac timing effect on RTs in cognitive tasks (Yang et al., 2017, 2023). Therefore, the precise association between BP status and cardiac timing effects remains unknown and needs further investigation.

Interoceptive processes regulating BP are under the control of the autonomic nervous system (ANS), which also plays a key role in integrating physiological states with cognitive function (Quadt et al., 2022; Thayer & Lane, 2000, 2009). According to the neurovisceral integration model, a dynamic interplay exists between higher‐order cortical regions and the ANS (Thayer & Lane, 2000, 2009). Parasympathetic activity, or vagal control, has been frequently examined in relation to the central autonomic network (CAN; Benarroch & Chang, 1993), due to the anatomical features of the vagus nerve and its neurotransmission (Thayer & Lane, 2009). As such, physiological indicators of vagal control may be related to embodied cognition. One widely used measure is vagally‐mediated heart rate variability (vmHRV), which reflects the influence of the vagus nerve on cardiac inter‐beat intervals and is considered an index of both CAN integrity and top‐down cognitive processes (Thayer et al., 2012; Thayer & Lane, 2000, 2009). Baroreceptor activation increases vagal input to the heart via the NTS, resulting in a positive correlation between baroreflex sensitivity and cardiac vagal control (La Rovere et al., 2008). Furthermore, greater cardiac vagal control, indicated by higher vmHRV, allows baroreceptors to receive more precise, real‐time cardiovascular information (Taylor & Eckberg, 1996). Thus, vmHRV may be linked to cardiac timing effects.

In addition to the CAN and baroreflex, vmHRV may influence cardiac timing effects through another pathway. Recent research suggests that cognitive control involved in complex cognitive tasks modulates the magnitude of cardiac timing effects (Adelhöfer et al., 2020; Larra et al., 2020; von Haugwitz et al., 2024; Yang, Herberlein, et al., 2024). Specifically, baroreceptor activation during cardiac systole has been shown to inhibit simple, automatic motor responses that require minimal cognitive effort, while it facilitates performance in complex tasks or dual‐task paradigms that demand substantial top‐down cognitive resources (Adelhöfer et al., 2020; Larra et al., 2020; Yang, Herberlein, et al., 2024). Moreover, vmHRV has been proposed as the indicator of top‐down recourses in cognitive performance (Spangler et al., 2022; Thayer et al., 2012; Yang et al., 2021, 2023, 2025). Therefore, it is possible that vmHRV would positively predict the magnitude of cardiac timing effects; however, this association may be attenuated in tasks that involve high levels of cognitive control. Despite the theoretical plausibility of this relationship, the extent to which vmHRV modulates cardiac timing effects remains unclear.

1.1

The current study aims to (1) examine whether cardiac timing effects are influenced by cognitive interference that imposes demands on top‐down regulatory resources; (2) investigate how blood pressure status and resting vmHRV are associated with cardiac timings under task conditions requiring varying levels of cognitive control. To assess cognitive performance, a computerized version of the Multi‐Source Interference Task (MSIT; Bush et al., 2003; Bush & Shin, 2006) was employed, with stimulus presentation time‐locked to the ECG R‐wave to manipulate cardiac timing. As a part of a larger project examining blood pressure variability, assessments of BP and HRV were conducted prior to experimental manipulations in separate sessions. This approach provided sufficient time for participants to adapt to the environment of physiological assessments and helped reduce potential state‐related confounds (e.g., room temperature, ambient light level, participants' expectations, and anxiety) in assessing trait‐level BP status (Weheida et al., 2017).

Based on the literature reviewed, we formulated the following hypotheses. First, we expected better cognitive performance during cardiac diastole compared to systole in the MSIT control trials, where low levels of cognitive control are required; however, the cardiac timing effect would diminish in the interference trials, which impose higher cognitive demands (Hypothesis 1). Given that the MSIT has also been employed as a laboratory stressor to elicit autonomic nervous system (ANS) reactivity (Gianaros et al., 2017; Sheu et al., 2012), we hypothesized a negative association between BP and cardiac timing effects in interference trials, with a weaker relationship in control trials (Hypothesis 2). Finally, we hypothesized that resting vmHRV would be positively associated with cardiac timing effects in control trials but that this association would be attenuated under the interference condition (Hypothesis 3).

## 2 METHOD

### 2.1 Participants

The sample for the current study consisted of 51 participants (age range = 18–43 years; *M* = 21.75 years; SD = 6.41 years; 35 female), who were recruited from undergraduate psychology courses. The sample size was estimated by the power analysis using the software G*Power 3.1.9 (Faul et al., 2009), in which the statistical significance level was set as α = 0.05, medium effect size *f* ^*2*^ = 0.15, and medium level power (1−*β*) = 0.8 (Cohen, 1988). All participants were right‐handed and nonsmokers and had no history of neurological or psychiatric disorders, cardiovascular disease, or other exclusion criteria, including current use of cardioactive or psychotropic medications. After recruitment, participants were instructed to abstain from alcohol for 24 h and from caffeine for 6 h prior to participation to ensure the validity of physiological data, in line with the recommendations of a recent study (Grant et al., 2023). Participants received course credits for their participation. Informed consent was obtained from participants prior to the study.

### 2.2 Blood pressure assessments

The blood pressure (BP) assessments in the current study are a part of a larger project that examines short‐term BP variability. All participants arrived at the laboratory for the BP assessments between 10:00 a.m. and 4:00 p.m., and the first BP measurement was taken after a 5‐min rest period. Six brachial BP measurements were taken while the participant was seated comfortably in a chair, with the torso upright, arms resting at heart level, and feet flat on the floor. Note that the number of BP measurements in the current study was higher than the minimal number (2 times) that was recommended to assess BP within a single visit (Muntner et al., 2019), which ensured the validity of the current BP assessments. A trained experimenter recorded systolic and diastolic BP (SBP and DBP), using an Omron Gold Wireless BP monitor (OMRON Corporation, Kyoto, Japan) on the participant's right upper arm. The interval between BP measurements was 7 min, during which the participants were instructed to relax and avoid excessive movement. The total duration of the BP assessment was approximately 40 min.

### 2.3 Heart rate variability measurements

To estimate short‐term resting heart rate variability (HRV), participants were instructed to sit still in a laboratory for 5 min. During the 5‐min resting period, an emotionally neutral film clip that depicted aquatic scenes were delivered on a desktop computer to maintain a relaxed but cognitively‐engaged mental state, which was consistent with “vanilla baseline” guidelines (Jennings et al., 1992). A BIOPAC MP160 system (BIOPAC Systems Inc, Goleta, CA) was used to collect physiological data, and raw signals were digitized at 1000 Hz (16‐bit) and analyzed with BIOPAC AcqKnowledge software 5.0 (BIOPAC Systems Inc., Goleta, CA). Electrocardiography (ECG) was recorded with disposable, pre‐gelled stress‐testing spot electrodes using a modified Lead II configuration (ConMed Andover Medical, Haverhill, MA). ECG R‐waves were labeled using the AcqKnowledge software and were then inspected manually by trained raters. All participants' ECG data met the criterion for further analyses where less than 5% of signals were influenced by artifacts. This criterion for the exclusion of an ECG epoch was made to follow the User's Guide of the Kubios software for HRV analysis (Biosignal Analysis and Medical Imaging Group, Kuopio, Finland), which is also consistent with the notion that abnormal beats and artifacts seriously distort HRV measures, and their correction should be limited to a low level (Berntson et al., 1997; Task Force, 1996). Additionally, respiratory activity was monitored by a respiration transducer that was placed around the participant's torso at the thoracic level.

### 2.4 Cognitive task, cardiac timing manipulation, and design

**FIGURE 1:**
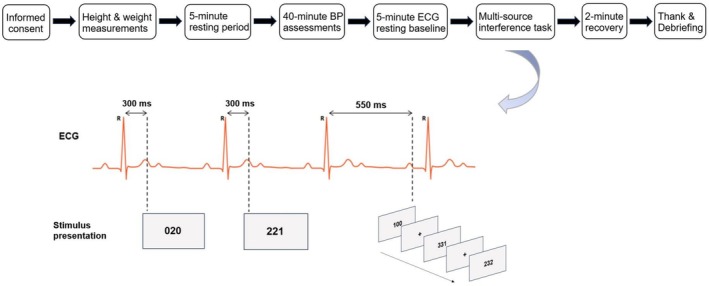
Procedure and stimulus presentation of the study. The exemplary trials of the Multi‐Source Interference Task indicated a control trial (“020”) and an interference trial (“221”) at cardiac systole (R + 300 ms), and the sequence of the trials included a control trial (“100”) and two interference trials (“331” and “232”) at cardiac diastole (R + 550 ms). Trials of all conditions were randomly counterbalanced in the task. BP, blood pressure; ECG, electrocardiography.

The Multi‐Source Interference Task (MSIT) was used to assess cognitive control (Bush et al., 2003). The MSIT integrates three dimensions of cognitively conflicting information that have been established in the Stroop (1935), Eriksen and Eriksen (1974), and Simon tasks (Simon & Berbaum, 1990), respectively (Bush et al., 2003, 2006). Specifically, in the MSIT, the participants were presented with a string of three digits as the task stimuli and were instructed to identify the “target” that differs from two other digits (“distractors”) by pressing the corresponding keys on a computer keyboard. The possible target digits included “1”, “2”, and “3”, while the digit “0” would only serve as the distractor in the task. The correct response to a target was to press the number key by a predefined finger of the dominant hand: pressing “1” using the index finger, “2” using the middle finger, and the “3” using the ring finger. As all participants were right‐handed, the location of the response keys was identical for all participants. The MSIT included two types of trials. In the *control trials*, the target digit was displayed in the location that matched the location of the response key, for example, “100”, “020”, and “003”. In the interference trials, the position of the target in the sequence did not match the location of the response key, for example, “211”, “322”, and “331” (see Figure 1).

In each MSIT trial, the stimuli were terminated by a detected response or were presented for 2500 ms if no key pressing was detected. The MSIT trials were presented at delay times of either 300 ms (cardiac systole) or 550 ms (cardiac diastole) from the ECG R‐wave. The timing of stimulus presentation was selected based on prior reports (e.g., Edwards et al., 2009; Schulz et al., 2009; Yang et al., 2023; Yang, Herberlein, et al., 2024). The visual stimuli were presented on a 61‐cm diagonal wide computer screen that was located 50 cm in front of the participant, using the E‐Prime 3.0 software (Psychology Software Tools, Pittsburgh, PA). R‐waves were detected online by an AccuSync 71 ECG trigger monitor (AccuSync, Milford, CT), which sent 5‐V TTL/CMOS compatible square waves to the E‐Prime software to trigger the stimulus presentation. The validity of the experimental settings to deliver the R‐wave‐locked stimuli was supported by our previous studies (Yang et al., 2019, 2023). The stimuli were scheduled to present during the first detected ECG R‐R interval that occurred 3500 ms after the end of the last trial. Thus, intertrial intervals (ITIs) were variable and ranged from 3500 ms to 4300 ms.

The MSIT included a total of 192 trials, including 96 control and 96 interference trials, respectively. In each trial type, 48 trials were delivered at cardiac systole and 48 trials at cardiac diastole. Therefore, the MSIT with cardiac timing manipulation constituted a 2 (trial type) × 2 (cardiac phase) full factorial within‐subject design. The MSIT trials were randomly counterbalanced (see Figure 1).

### 2.5 Procedure

Upon arrival at the laboratory, informed consent was obtained from all participants. Demographic information was then collected using a questionnaire. Before the experiment began, participants' height and weight were measured to calculate their body mass index (BMI). They were then comfortably seated in the laboratory for 5 min, after which blood pressure assessments were conducted. Next, physiological sensors, including ECG electrodes and a respiration transducer, were attached for a resting baseline period. During the resting baseline, participants were instructed to sit quietly and comfortably and breathe normally for 5 min. At the end of the baseline measurement, the respiration transducer was removed, and the ECG electrodes were reconnected to the AccuSync trigger monitor. The experimenter then instructed participants to perform the Multi‐Source Interference Task (MSIT) and complete 10 practice trials. During the instructions and practice phase, participants were encouraged to ask any questions. Once participants indicated they were ready, the experimenter exited the laboratory room, and the MSIT began. The cognitive task lasted approximately 10 min. Following the task, participants were asked to sit still and remain quiet for 2 min during a recovery period. Finally, the physiological recording sensors were removed, and participants were thanked and debriefed about the purpose of the study (see Figure 1).

### 2.6 Data reduction

Blood pressure status was assessed using mean systolic and diastolic blood pressure (SBP and DBP), calculated as the average of six SBP and DBP readings, respectively. As the cardiac timing effects were thought to reflect baroreceptor activation, SBP was entered into the regression models as the index of BP status (see the later sections).

For ECG data, inter‐beat intervals (IBIs) were extracted from the ECG recordings obtained during the resting baseline. Heart rate variability (HRV) was analyzed using both time‐ and frequency‐domain measures with Kubios HRV analysis software 2.0 (Biosignal Analysis and Medical Imaging Group, Kuopio, Finland). Specifically, mean IBI length, the root mean square of successive differences (RMSSD) between R‐R intervals, and the percentage of successive R‐R intervals differing by more than 50 ms (pNN50) were calculated as time‐domain HRV indices (Berntson et al., 1997; Task Force, 1996). In the frequency domain, high‐frequency HRV (HF‐HRV; 0.15–0.4 Hz), low‐frequency HRV (LF‐HRV; 0.04–0.15 Hz), and the LF/HF ratio were derived using fast Fourier transform spectral analysis as frequency‐domain HRV indices. Due to their skewed distributions, HF‐HRV and LF‐HRV values were log‐transformed using the natural logarithm (base *e*) to normalize the data. Respiration data were used to detect gross respiratory artifacts in the ECG signal and to validate the selection of frequency bands for HRV analysis. Although there is a general agreement that most HRV metrics inform on parasympathetic activity (Reyes del Paso et al., 2013; Shaffer & Ginsberg, 2017), HF‐HRV has been used as the primary indicator of cardiac vagal control (Thayer et al., 2012; Thayer & Lane, 2009). Therefore, HF‐HRV was entered into the regression models for further analyses (see the later sections).

Behavioral performance on the MSIT included measures of response accuracy and response time (RT). Response accuracy was calculated as the percentage of correct responses across all trials for each condition. Mean RTs were computed for correct trials within each condition. RT outliers were defined as trials with RTs outside ±3 standard deviations from the mean RT for each condition or RTs shorter than 100 ms, the latter indicating potential guessing‐related responses. These outliers accounted for 1.1% of all correct MSIT trials and were excluded from analysis, consistent with the recommendation by Ratcliff (1993).

### 2.7 Analytic approach

Relationships among variables were analyzed using Pearson correlations. To examine the effects of cognitive interference and cardiac timing on behavioral performance, response accuracy and mean RTs were submitted to 2 (trial type) × 2 (cardiac phase) repeated measures analyses of covariance (ANCOVA). Note that gender is known to influence cardiac timing effects (Yang, Herberlein, et al., 2024; Yang, Chaney, et al., 2024), which were controlled for in the analyses as the covariate.

Separate multiple regression models were constructed to investigate the influence of BP status and cardiac vagal control on cardiac timing effects on the interference and control trials. The equation of the regression models was the same for the two trial types:

$$\begin{array}{c} \mathrm{CTE}=\beta_{0}+\beta_{1}\mathrm{SBP}+\beta_{2}\mathrm{HF}-\mathrm{HRV}+\beta_{3}\text{Gender}\\ +\beta_{4}\mathrm{IBI}+\beta_{5}\text{Accucacy}+\beta_{6}\text{Mean}\;\mathrm{RT} \end{array}$$

In the equation, cardiac timing effect (CTE) on RT represents the RT change score between cardiac phases: RT_systole_ – RT_diastole_, which was entered into the model as the dependent variable. The interference trials and the control trials were examined in separate regression models. SBP and HF‐HRV were independent variables and were mean‐centered in the regression models. The coefficients β_0_, β_1_, and β_2_ represent the intercept and the effects of the independent variables, respectively. Gender, resting IBI, response accuracy, and mean RT in each condition might influence cardiac timing effects and were thus entered into the models as covariates; and β_3_–β_6_ are the coefficients of the covariates. All participants were binary gender, which was entered into the model using dummy coding, and female was the reference level. In addition, RMSSD was entered into the regression models as a substitute vmHRV measure to check the validity of HF‐HRV in reflecting cardiac vagal control. HF‐HRV was chosen as the primary indicator of vmHRV due to that (1) RMSSD was recommended as a descriptive measure of HRV (Task Force, 1996) but it may not optimally indicate the periodic patterning that characterizes HRV (Quigley et al., 2024); and (2) RMSSD is influenced by frequencies outside breathing frequencies and may not serve as the primary index of phasic parasympathetic activity induced by cardiorespiratory coupling (Quigley et al., 2024). In the present study, the average respiratory frequency was 0.29 Hz (SD = 0.05), ranging from 0.19 to 0.40 Hz (see Table 1), which falls within the defined frequency band for HF‐HRV. In addition, RMSSD was highly correlated with HF‐HRV, *r* = 0.84, *p* < 0.01 (see Table 2).

**TABLE 1:** Descriptive statistics by genders.

Variable	Female (*n* = 35)	Male (*n* = 16)	*t*‐statistic
Age (*M* years, SD)	21.89 (6.61)	21.44 (6.15)	0.23
Body mass index (*M*, SD)	26.95 (5.32)	25.82 (6.82)	0.65
Systolic BP (*M* mm Hg, SD)	109.43 (10.43)	123.08 (13.23)	−4.15***
Diastolic BP (*M* mm Hg, SD)	73.90 (8.18)	77.57 (8.03)	−1.50
Resting IBI length (*M* ms, SD)	791.83 (119.48)	828.27 (110.20)	−1.04
RMSSD (*M* ms, SD)	51.40 (30.61)	43.24 (17.79)	0.99
pNN50 (*M* %, SD)	28.33 (21.32)	20.00 (13.55)	1.67
HF‐HRV (*M* ln ms^2^, SD)	6.71 (1.44)	6.60 (0.86)	0.30
LF‐HRV (*M* ln ms^2^, SD)	6.56 (1.13)	6.93 (1.29)	−1.05
LF/HF ratio (*M*, SD)	1.14 (0.90)	1.58 (1.03)	−1.54
Respiratory rate (*M* bpm, SD)	17.40 (3.15)	16.83 (3.33)	0.58
Response accuracy (*M* %, SD)			
Interference			
Systole	90.77 (12.50)	96.22 (4.45)	−1.69
Diastole	91.07 (12.36)	94.92 (7.01)	−1.16
Control			
Systole	99.57 (0.86)	98.54 (3.94)	1.04
Diastole	99.71 (0.72)	98.60 (3.55)	1.24
Response time (*M* ms, SD)			
Interference			
Systole	996.17 (187.72)	890.43 (105.22)	2.57*
Diastole	988.47 (194.54)	882.70 (110.07)	2.03*
Control			
Systole	587.80 (164.08)	510.92 (72.72)	2.32*
Diastole	572.25 (153.83)	496.34 (59.67)	2.54*

*Note*: The Multi‐Source Interference Task performance was assessed as response accuracy and response time in different types of trials and cardiac phases.

Abbreviations: BP, blood pressure; HF‐HRV, high‐frequency heart rate variability; IBI, inter‐beat interval; LF‐HRV, low‐frequency heart rate variability; pNN50, the percentage of successive R‐R intervals differing by more than 50 ms; RMSSD, root mean square of successive differences.

* *p* < 0.05.

*** *p* < 0.001.

**TABLE 2:** Pearson correlation coefficients among variables.

Variable	1	2	3	4	5	6	7	8	9	10	11	12
1. Age	—											
2. Body mass index	0.22	—										
3. Systolic BP	−0.08	0.39**	—									
4. Diastolic BP	0.21	0.59**	0.66**	—								
5. Resting IBI	−0.04	−0.12	0.13	−0.03	—							
6. RMSSD	−0.35*	−0.09	−0.03	−0.21	0.62**	—						
7. pNN50	−0.39**	−0.02	−0.08	−0.13	0.54**	0.91**	—					
8. HF‐HRV	−0.39**	0.02	0.02	−0.03	0.49**	0.84**	0.86**	—				
9. LF‐HRV	−0.22	−0.19	0.08	−0.05	0.47**	0.61**	0.50**	0.56**	—			
10. LF/HF ratio	0.11	−0.22	−0.01	−0.15	−0.18	−0.27	−0.39**	−0.51**	0.23	—		
11. Respiration rate	0.21	0.39**	−0.10	0.24	−0.17	−0.10	−0.12	−0.10	0.07	0.09	—	
12. MSIT accuracy	0.07	−0.15	−0.19	−0.24	−0.18	−0.07	−0.04	−0.10	0.02	0.17	0.10	—
13. MSIT RT	−0.08	−0.16	−0.18	−0.21	−0.37**	−0.32*	−0.31*	−0.45**	−0.40**	0.16	−0.11	−0.02

*Note*: Accuracy and response time (RT) of the Multi‐Source Interference Task (MSIT) were averaged across all conditions.

Abbreviations: BP, blood pressure; HF‐HRV, high‐frequency heart rate variability; IBI, inter‐beat interval; LF‐HRV, low‐frequency heart rate variability; pNN50, the percentage of successive R‐R intervals differing by more than 50 ms; RMSSD, root mean square of successive differences.

* *p* < 0.05.

** *p* < 0.01.

*** *p* < 0.001.

Statistical significance was tested with an alpha of 0.05. Effect sizes of the factors in the ANCOVA and the independent variables in the regression models were estimated with partial eta‐squared and *R* ^*2*^, respectively.

## 3 RESULTS

### 3.1 Descriptive statistics

For the full sample (*N* = 51), the mean body mass index (BMI) was 26.60 (SD = 5.73). The average systolic blood pressure (SBP) was 113.91 mm Hg (SD = 5.73), and the average diastolic blood pressure (DBP) was 75.05 mm Hg (SD = 5.73). Regarding cardiac measures, the mean inter‐beat interval (IBI) was 803.26 ms (SD = 116.80); mean RMSSD was 48.79 ms (SD = 27.25); mean pNN50 was 25.66% (SD = 19.43%); mean high‐frequency HRV (HF‐HRV) was 6.67 ln ms^2^ (SD = 1.27); mean low‐frequency HRV (LF‐HRV) was 6.68 ln ms^2^ (SD = 1.18); and the mean LF/HF ratio was 1.28 (SD = 0.95).

Following the recommendation of the American Heart Association (Gulamhusein et al., 2024), the gender‐stratified demographical, physiological, and behavioral measurements are displayed in Table 1. Compared to female participants, male participants exhibited higher systolic blood pressure and faster RTs. However, gender did not influence other variables (see Table 1).

As shown in Table 2, BMI was positively correlated with BP measures and respiratory rate, and there were positive correlations among cardiac measures. Of note, longer IBI and higher levels of HRV measures were associated with shorter RT across all trials (see Table 2), indicating resting cardiac vagal control predicted faster response speed.

### 3.2 Cardiac timing effects on cognitive performance

**FIGURE 2:**
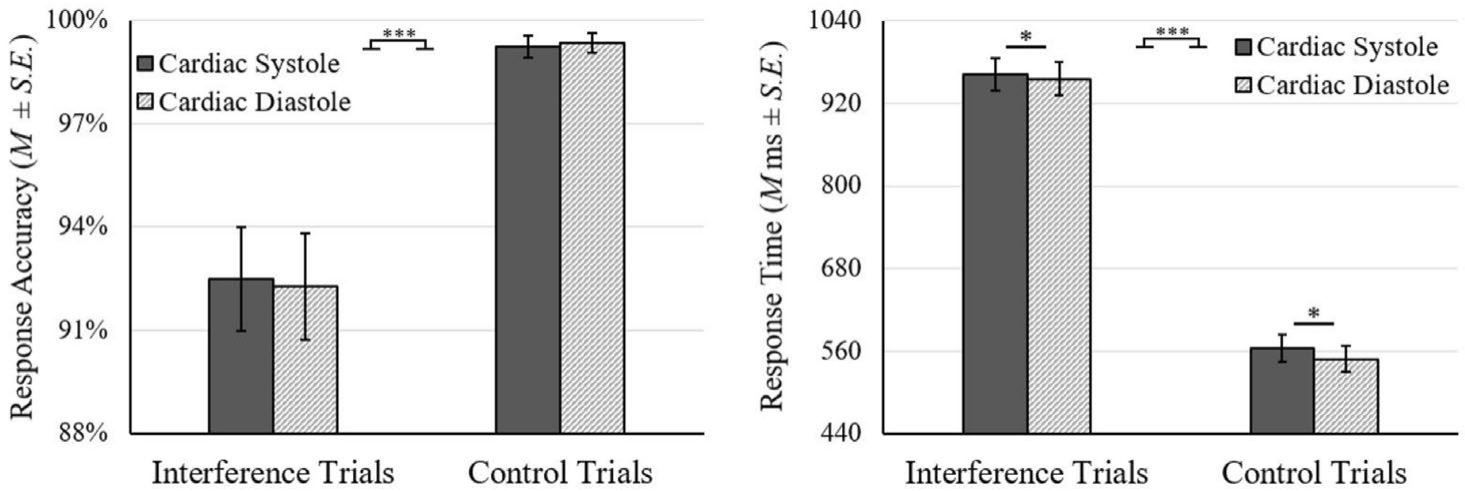
Cardiac timing effects on behavioral performance of Multi‐Source Interference Task. *p < 0.05; ***p < 0.001.

The effects of cardiac timing and cognitive interference were examined by repeated measures ANCOVA, in which gender was the covariate. The results of ANCOVA on mean response accuracy showed a main effect of trial type, *F* (1, 49) = 24.23, *p* < 0.001, $\eta_{p}^{2}$ = 0.331. However, there was no main effect of cardiac phase, *F* (1, 49) = 0.38, *p* = 0.542, $\eta_{p}^{2}$ = 0.002, nor interaction between trial type and cardiac phase, *F* (1, 49) = 0.06, *p* = 0.808, $\eta_{p}^{2}$ = 0.001 (see Figure 2). Therefore, response accuracy in the MSIT was reduced by cognitive interference but not influenced by the cardiac timing of stimulus presentation.

As for mean RT, repeated measures ANOVA indicated main effects of trial type, *F* (1, 49) = 453.26, *p* < 0.001, $\eta_{p}^{2}$ = 0.902, and cardiac phase, *F* (1, 49) = 6.82, *p* = 0.012, $\eta_{p}^{2}$ = 0.122. However, the interaction between trial type and cardiac phase was not evident, *F* (1, 49) = 0.74, *p* = 0.394, $\eta_{p}^{2}$ = 0.002 (see Figure 2). The result suggests that RT was prolonged by cognitive interference and when MSIT stimuli were presented during cardiac systole.

### 3.3 Differential effects of blood pressure and vagal control on cardiac timing effects

**FIGURE 3:**
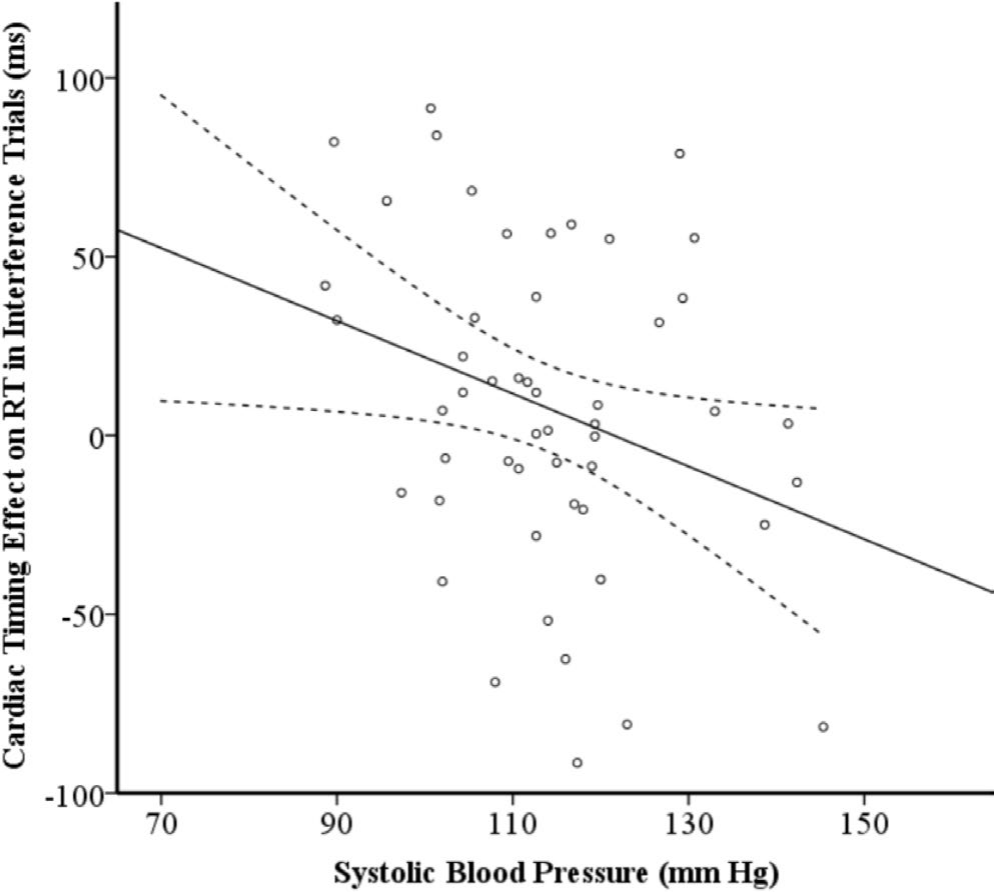
The scatter plot of the association of the cardiac timing effect with systolic blood pressure in interference trials. The cardiac timing effect was calculated as the difference score in reaction time (RT) between cardiac systole and diastole. The solid line represents the regression line of the association, and the dashed lines indicate the 95% confidence interval.

The multiple regression analyses are summarized in Table 3. Model 1 tested the effects of SBP and HF‐HRV on cardiac timing effects on RT in MSIT interference trials. The results of Model 1 showed that lower SBP predicted larger cardiac timing effects on RT, β_1_ = −1.30, 95% CI = −2.42 to 0.18, *p* = 0.024, *R* ^2^ = 0.10, but HF‐HRV was not related to the dependent variable (see Table 3 and Figure 3).

**TABLE 3:** Summary of the multiple regression models.

Independent variable/*covariates*	Model 1 (interference trials)		Model 2 (control trials)	
	β (*SE*)	*R* ^*2*^	β (*SE*)	*R* ^*2*^
Systolic blood pressure	−1.30 (0.56)*	0.10	−0.60 (0.33)	0.06
HF‐HRV	−5.27 (6.00)	0.01	−6.49 (3.67)	0.05
*Gender*	6.37 (16.24)	0.01	8.84 (9.54)	0.01
*Resting IBI*	−0.03 (0.06)	0.02	0.09 (0.04)*	0.05
*Response accuracy*	121.60 (98.64)	0.08	16.22 (183.72)	0.01
*Mean reaction time*	−0.08 (0.04)	0.02	0.07 (0.03)*	0.13

*Note*: The dependent variable in both models was the cardiac timing effect on reaction time (RT), which was calculated as (RT_systole_−RT_diastole_). The independent variables, systolic blood pressure and HF‐HRV, were mean‐centered in the models; the coefficients in the table are unstandardized. Both models were controlled for gender, resting IBI, response accuracy, and mean reaction time in each condition (serving as the baseline response speed), and all covariates were displayed in italicized fonts.

Abbreviations: HF‐HRV, high frequency heart rate variability; IBI, inter‐beat interval.

*p < 0.05.

In contrast, Model 2 indicated that marginal trends to negative associations of cardiac timing effects of RTs in control trials with SBP, β_1_ = −0.60, *p* = 0.078, *R* ^2^ = 0.06, and with HF‐HRV, β_2_ = −6.49, *p* = 0.083, *R* ^2^ = 0.05 (see Table 3).

To validate the regression models, RMSSD was entered into the models as the substitute vmHRV measure. The results of RMSSD and the original Model 1 and 2 were similar. Specifically, SBP was negatively related to cardiac timing effects in Model 1, β_1_ = −1.30, 95% CI = −2.44 to –0.15, *p* = 0.027, *R* ^2^ = 0.10, while RMSSD was not a significant predictor in Model 1, β_2_ = −0.27, *p* = 0.41. In Model 2, SBP and RMSSD only marginally predicted cardiac timing effects in control trials: β_1_ = −0.60, *p* = 0.082, *R* ^2^ = 0.06; β_2_ = −0.32, *p* = 0.094, *R* ^2^ = 0.05 (see Table S1). The results of the models with RMSSD were consistent with those of the models with HF‐HRV.

## 4 DISCUSSION

In the present study, we investigated how cardiac timing effects on cognitive performance were associated with blood pressure (BP) status and vagally‐mediated heart rate variability (vmHRV) in healthy young adults. Cognitive interference was manipulated using the Multi‐Source Interference Task (MSIT), and cardiac timing was controlled via R‐wave–locked stimulus presentation. To minimize potential confounding effects from physiological and mental states, cardiac timing effects and trait‐level BP and HRV were assessed in separate sessions. A comprehensive set of HRV metrics was collected, and relevant covariates were statistically controlled in the analyses. Our hypotheses regarding task performance and BP status (H1 and H2) were partially supported. However, the hypothesis about vmHRV (H3) was not supported by the results. Although no interaction was observed between MSIT trial type and cardiac phase, cardiac timing effects on response time (RT) were present in both interference and control trials. Notably, the main findings revealed that systolic BP was negatively associated with cardiac timing effects during interference trials, whereas vmHRV was not related to cardiac timing effects during interference or control trials. These results offer direct evidence that individual differences in autonomic nervous system (ANS) functioning modulate cardiac timing effects on cognitive performance.

The first hypothesis regarding cardiac timing effects on cognitive performance was partially supported. In line with previous findings (Birren et al., 1963; Callaway & Layne, 1964; Edwards et al., 2007, 2009; Yang et al., 2017, 2023), sensorimotor processing in control trials was faster during cardiac diastole compared to systole. However, contrary to our prediction, cognitive interference did not attenuate or reverse the cardiac timing effect in interference trials. Prior studies have demonstrated that cardiac timing effects can interact with higher‐order cognitive processes and may even be reversed in tasks involving elevated working memory load (Yang, Herberlein, et al., 2024) or cognitive conflicts (Adelhöfer et al., 2020; Larra et al., 2020). Several mechanisms may explain how cognitive demands modulate cardiac timing effects. First, as suggested by Larra et al. (2020), residual neural activation in response to sensory input may diminish the modulatory influence of baroreceptor afferents. Second, the central processing of external stimuli under cognitive load may override interoceptive signals that underlie cardiac timing effects (Adelhöfer et al., 2020). Third, complex response demands, such as conflict monitoring and inhibitory control, may prolong motor responses, resulting in a misalignment between the timing of stimulus presentation and the phase of cardiac response execution (Yang, Herberlein, et al., 2024).

The present findings did not support any of the previously proposed explanations but instead suggest a possible additive effect, that is, cardiac timing effects might act additively across the serial stages of sensorimotor processing (Sanders, 1980). Put another way, cortical inhibition elicited by baroreceptor activation may not be overridden by top‐down cognitive control. Rather, cardiac timing effects may influence one or more stages of sensorimotor processing, and these effects are likely to manifest in behavioral measures regardless of whether responses rely primarily on automatic or controlled processes. The inconsistency between the current results and prior studies (Adelhöfer et al., 2020; Larra et al., 2020; Yang, Herberlein, et al., 2024) may reflect differences in the speed–accuracy trade‐off across tasks. However, there are limitations in that account for the absence of an interaction between MSIT condition and cardiac phase. For example, small effect sizes and low statistical power may obscure interactive effects (Gelman & Stern, 2006; Nieuwenhuis et al., 2011). Therefore, the null finding of an interaction could not serve to support a strong conclusion about cognitive processes (Loftus, 1978). Future research should explore cardiac timing effects on internal cognitive operations using computational modeling approaches to better understand these dynamics.

The second hypothesis about the relationship between BP status and cardiac timing effects was supported by the results. The MSIT interference trials are known to elevate BP and activate brain regions involved in interoception and BP regulation (Bush et al., 2003; Gianaros et al., 2017; Sheu et al., 2012), thereby serving as a cognitive stressor. Consistent with previous findings (Schulz et al., 2011), we observed that cognitive stress modulated cardiac timing effects: higher systolic BP (SBP) was associated with reduced cardiac timing effects in interference trials. Although resting BP and BP reactivity to stress are distinct constructs, they are interrelated and jointly contribute to cardiovascular disease (CVD) risk (Gianaros et al., 2017). In contrast, BP was not associated with cardiac timing effects in control trials that lacked cognitive interference or stress, suggesting that in subclinical populations, disruptions in baroreflex function may only emerge under stress. These findings imply that altered BP reactivity could precede clinically measurable elevations in BP.

The third hypothesis regarding the association of vmHRV with cardiac timing effects was not supported. Surprisingly, vmHRV was not associated with the magnitude of the cardiac timing effect on RTs in interference or control trials. As vmHRV is thought to serve as an index of top‐down cognitive resources (Thayer et al., 2012; Thayer & Lane, 2009), the presence of cognitive interference may have engaged those resources, thereby diminishing any relationship between vmHRV and performance differences across cardiac cycle phases in the MSIT interference condition. However, the null finding of control trials appears to contradict previously reported positive associations between vagal control and both baroreflex sensitivity (La Rovere et al., 2008; Taylor & Eckberg, 1996) and cognitive functioning (Arakaki et al., 2023; Forte et al., 2019). However, a closer examination of RTs across cardiac phases revealed that responses were faster during both systole and diastole, aligning with the observed negative correlations between HRV measures and overall RT (see Table 2). In other words, individuals with greater cardiac vagal control tend to respond more quickly overall, and this general speeding may reduce the observable cardiac timing effect, as indicated by the significant effect of baseline RT on the cardiac timing effect in Model 2 (see Table 3). An alternative explanation for the unexpected vmHRV findings involves gender differences in autonomic nervous system (ANS) functioning. Compared to men, women tend to exhibit higher levels of cardiac vagal control (Koenig & Thayer, 2016), and prior work has also shown that cardiac timing effects are positively associated with resting vmHRV in males, but not in females (Yang, Chaney, et al., 2024). In the current study, over two‐thirds of participants were female, suggesting that the anticipated association between vmHRV and RTs in control trials may have been obscured by the distinct physiological patterns observed in female participants.

### 4.1 Implications

The findings of the present study have both theoretical and practical implications. As demonstrated by our results, cardiac timing effects represent a heterogeneous set of phenomena. Baroreceptor‐driven interoceptive processes appear to influence different domains of cognition in distinct ways. While information processing is generally enhanced during cardiac diastole, certain cognitive functions may be more efficient during systole. For example, in affective and social contexts, baroreceptor activation has been shown to facilitate impulsive decision‐making (Kimura et al., 2023), enhance fear learning (Pfeifer et al., 2017), and improve both emotional memory recall (Fiacconi et al., 2016) and threat detection (Azevedo et al., 2017). These findings emphasize the role of interoception in encoding environmental information, with the selective enhancement of fear processing interpreted as an adaptive survival mechanism (Critchley & Garfinkel, 2015).

This line of research differs from the present study in a key respect: whereas those prior studies above have focused on how interoceptive signals shape the mental representation of affective stimuli, our study was primarily concerned with the real‐time, dynamic “online” influence of interoceptive processes on cognitive performance.

Moreover, our findings support that cardiac timing effects may vary depending on contextual factors such as task demands, mental workload, and the degree of cognitive control required. These variations result in differing directions and magnitudes of cardiac timing effects, as well as distinct associations with physiological variables. A recent review by Caparco et al. (2025) questioned the validity of methods that synchronize stimulus presentation with cardiac phases. While prior research has generally supported the reliability of such manipulations, the concerns raised in the review are not unfounded. A variety of factors in research subjects, such as gender, IBI length, BP status, and ANS function integrity, can influence the effectiveness of cardiac timing paradigms. In the present study, we accounted for these variables and found that they impacted the cardiac timing effects observed with time‐locked stimulus presentation. Accordingly, future research should adopt a more comprehensive approach that considers both task‐related and trait‐level factors.

## 5 LIMITATIONS, FUTURE DIRECTIONS, AND CONCLUSION

Our findings should be interpreted in light of several limitations. First and foremost, the study did not include continuous beat‐to‐beat blood pressure (BP) measurements. As a key indicator of baroreflex sensitivity, beat‐to‐beat BP will help validate the cardiac timing manipulation and provide a clearer understanding of the interoceptive mechanisms underlying cardiac timing effects. Second, the present study lacked direct neural measures of information processing. Incorporating techniques such as event‐related potentials (ERPs) or neuroimaging in future research will offer valuable insights into the neural mechanisms and temporal dynamics underlying cardiac timing effects. Third, our sample predominantly consisted of female participants, and our findings should be replicated with gender‐balanced samples. Relatedly, all participants were healthy young adults with no history of CVD, limiting the generalizability of our findings. Future studies should aim to replicate these results in more diverse and clinically relevant populations. Last but not least, there was no check for the accuracy of the timing of stimulus presentation. Although the system used in the present study has been validated in the previous studies (Yang et al., 2019, 2023), it is important to add the manipulation check for the R‐wave‐locked stimulus presentation in future studies.

In sum, the present study is among the first to examine how trait‐level physiological characteristics influence cardiac timing effects. We employed clinically standardized procedures to assess blood pressure and cardiac metrics, utilized a well‐established cognitive stressor, controlled for key covariates in our analyses, and validated the findings using an alternative vmHRV measure. Despite a few weaknesses, the methodological features of the present study allow for the investigation of important factors influencing cardiac timing effects. Our results offer valuable insights for future research on cardiac timing effects and will contribute to a deeper understanding of how interoceptive processes shape human cognition.

## FUNDING INFORMATION

The study was supported by the American Psychological Foundation 2024 F.J. McGuigan Early Career Investigator Research Grant that was awarded to the leading author, Dr. Xiao Yang.

## CONFLICT OF INTEREST STATEMENT

There are no conflicts of interest.

## ETHICS STATEMENT

This study was conducted in accordance with the Declaration of Helsinki and approved by the ODU Institutional Review Board. Informed consent was obtained from all subjects involved in this study.

## Supporting information


Table S1.

## Data Availability

The raw data supporting the conclusions of this article will be made available by the authors on request.
