# Supplementary material for: Cardiac timing effects on response speed are modulated by blood pressure but not heart rate variability in healthy young adults
Source: Physiol Rep. 2025 Sep 29;13(19):e70590. doi: 10.14814/phy2.70590 (PMC12477440; doi:10.14814/phy2.70590)
Supplement: Supplementary file 1 — Table S1. [file PHY2-13-e70590-s001.docx]

**Supplemental Materials**

**Table 1. Summary of the Multiple Regression Models with RMSSD**

| Independent Variable/*Covariates* | **Model 1**  (Interference Trials) | | |  | | **Model 2**  (Control Trials) | | |
| --- | --- | --- | --- | --- | --- | --- | --- | --- |
|  | β (*SE*) | *R^2^* | |  | β (*SE*) | | *R^2^* | |
| Systolic Blood Pressure | -1.30 (0.57)* | | 0.10 |  | | -0.60 (0.34) | | 0.06 |
| RMSSD | -0.27 (0.32) | | 0.01 |  | | -0.32 (0.19) | | 0.05 |
| *Gender* | 4.26 (17.50) | | 0.01 |  | | 7.09 (10.10) | | 0.01 |
| *Resting IBI* | -0.02 (0.07) | | 0.02 |  | | 0.11 (0.04)* | | 0.05 |
| *Response Accuracy* | 130.30 (99.59) | | 0.08 |  | | 26.78 (187.13) | | 0.01 |
| *Mean Reaction Time* | -0.08 (0.04) | | 0.02 |  | | 0.08 (0.03)** | | 0.12 |

*Note*: RMSSD = the root mean square of successive differences; IBI = inter-beat interval. The dependent variable in both models was Cardiac Timing Effect on reaction time (RT), which was calculated as (RT_systole_ – RT_diastole_). The independent variables, systolic blood pressure and HF-HRV, were mean-centered in the models; the coefficients in the table are unstandardized. Both models were controlled for gender, resting IBI, response accuracy, and mean reaction time in each condition (serving as the baseline response speed); and all covariates were displayed in italicized fonts.

**p* < .05; **p* < .01

**Table 2. Summary of the Linear Mixed Models**

| Independent Variable/*Covariates* | **Model 1**  (with HF-HRV) | | | |  | | | **Model 2**  (with RMSSD) | | |
| --- | --- | --- | --- | --- | --- | --- | --- | --- | --- | --- |
|  | β (*SE*) | *p*-value | | |  | | β (*SE*) | | *p*-value | |
| Systolic Blood Pressure | **-0.38 (0.15)** | | **.014** |  | | **-0.37 (0.15)** | | | | **.019** |
| vmHRV indicator | -0.19 (0.15) | | .224 |  | | -0.14 (0.17) | | | | .404 |
| Trial Type | -0.18 (0.22) | | .425 |  | | -0.18 (0.23) | | | | .433 |
| Systolic Blood Pressure × Trial Type | 0.18 (0.20) | | .370 |  | | 0.17 (0.20) | | | | .397 |
| vmHRV × Trial Type | 0.02 (0.20) | | .910 |  | | -0.03 (0.20) | | | | .867 |
| *Gender* | 0.33 (0.25) | | .192 |  | | 0.26 (0.27) | | | | .340 |
| *Resting IBI* | 0.01 (0.01) | | .190 |  | | 0.01 (0.01) | | | | .193 |
| *Response Accuracy* | 2.28 (2.08) | | .277 |  | | 2.45 (2.12) | | | | .250 |

*Note*: HF-HRV = high frequency heart rate variability; RMSSD = the root mean square of successive differences; vmHRV = vagally-mediated heart rate variability; IBI = inter-beat interval. The type of Multi-source Interference Task trials was entered as a factor in the linear mixed models, in which the dependent variable was Cardiac Timing Effect on reaction time (RT), which was calculated as the ratio between RT_systole_ and RT_diastole_ (**RT_systole_/RT_diastole_**). This calculation removed the effects of baseline RT on the cardiac timing effect. The independent variables included Systolic Blood Pressure, vmHRV, Trial Type (using dummy coding, and control condition served as the reference), and the interactions of Trial Type with blood pressure and vmHRV. The indicators of vmHRV in Model 1 and 2 are HF-HRV and RMSSD, respectively. In both models, covariates included gender, resting IBI, and response accuracy, which were displayed in italicized fonts. The coefficients in the table are unstandardized. Systolic blood pressure was the only significant predictor of the ratio between RT_systole_ and RT_diastole_ in both models.
